# Association of selected genetic variants in *CB*S and *MTHFR* genes in a cohort of children with homocystinuria in Sri Lanka

**DOI:** 10.1186/s43141-022-00449-7

**Published:** 2022-12-13

**Authors:** Nadeesha Samarasinghe, Dinithi Mahaliyanage, Sumadee De Silva, Eresha Jasinge, Nimal Punyasiri, H. W. Dilanthi

**Affiliations:** 1 Institute of Biochemistry, Molecular Biology and Biotechnology, University of Colombo, Colombo 03, Sri Lanka; 2 Lady Ridgeway Hospital for Children, Colombo, Sri Lanka; 3 Department of Biochemistry and Molecular Biology, Faculty of Medicine, University of Colombo, Colombo 08, Sri Lanka

**Keywords:** Homocystinuria, Methylenetetrahydrofolate reductase, Cystathionine β-synthase

## Abstract

**Background:**

Homocystinuria is an inherited, inborn error of homocysteine metabolism, which leads to the abnormal accumulation of homocysteine and its metabolites in blood and urine, resulting in various complications. Variants in the *cystathionine β-synthase (CBS)* and *methylenetetrahydrofolate reductase (MTHFR)* genes interrupt the formation of the corresponding enzymes and prevent homocysteine from being metabolised; hence, the homocysteine levels in plasma increase than the optimum levels.

**Materials and methods:**

In the current study, eight clinically confirmed children with homocystinuria were detected to study the chosen variants in the *CBS* gene (c.833 T>C and c.19del) and in the *MTHFR* gene (c.665 C>T, c.1286 A>C) using SNaPshot mini-sequencing and direct sequencing.

**Results:**

After screening eight patients, none had the c.833T>C, but four patients were in the homozygous state for the c.19del variant in the *CBS* gene. Furthermore, seven were heterozygous for c.1286A>C, while one patient was heterozygous for c.665C>T in the *MTHFR* gene.

**Conclusion:**

According to the results, c.19del is common in the studied cohort of Sri Lankan children, while c.833T>C is absent, whereas c.1286A>C was more frequent than c.665C>T. To our knowledge, the current study was the first report to discuss the genetic impact of homocystinuria in Sri Lanka; further comprehensive studies are necessary with a larger sample size to establish the association of these variants with the disease in Sri Lanka, which can be beneficial in enhanced patient care and for prospective studies.

## Background

Homocystinuria is an inborn metabolism error and an autosomal recessive inherited disorder in the transsulfuration or methylation pathway, which leads to high plasma/urinary levels of homocysteine and methionine and low level of cystathionine and cysteine. A genetic defect in one of the enzymes (*CBS*—cystathionine β-synthase, *MTHFR*—methylenetetrahydrofolate reductase, *MTR*—5-methyltetrahydrofolate-homocysteine methyltransferase, *MTRR*—5-methyltetrahydrofolate-homocysteine methyltransferase reductase) in homocysteine metabolism may lead to metabolic disruption. In addition, nutritional deficiency of one or more of the cofactors (vitamins B2, B6, B12, and folate) in the homocysteine metabolism is also causing metabolic disruption. As a result, homocysteine levels are increased (hyperhomocysteinemia) [1], and it can be confirmed by measuring plasma total homocysteine levels in the blood.

The most common form of homocystinuria is caused by a deficiency in the production of cystathionine β-synthase (CBS) enzyme (OMIM no. 236200). This enzyme catalyses the first committed step of transsulfuration by conjugating homocysteine and serine to form cystathionine and then subsequently converting into cysteine and 𝛼-ketoglutarate [2]. The human *CBS* gene contains 17 exons and is located on chromosome 21q22.3 [3], encoding 552 amino acid length CBS enzyme, which assembles into a homo-tetrameric protein with 63 kDa subunits [4]. Variants in the *CBS* gene interrupt the CBS enzyme production, preventing homocysteine from being further metabolised. Thus, homocysteine levels increase in blood, and excess is excreted in the urine. According to the Human Gene Mutation Database, in 2022, nearly 200 different genetic variants are reported in the *CBS* gene [5].

Furthermore, the variants in the *MTHFR* gene could interfere with the methylenetetrahydrofolate reductase enzyme (MTHFR) production (OMIM no. 236250), disrupting the conversion of folic acid into 5-methyltetrahydrofolate, which in turn is used as the methyl donor in the remethylation pathway when converting homocysteine into methionine, leading to the interruption of methionine formation from homocysteine. However, homocysteine forms methionine in an uninterrupted remethylation pathway by adding a methyl group from 5-methyltetrahydrofolate, which is formed when folic acid is converted into 5-methyltetrahydrofolate by the MTHFR enzyme. The human *MTHFR* gene contains 12 exons, and it is located on chromosome 1p36.3. The gene encodes MTHFR protein, a homodimer consisting of an N-terminal catalytic domain in each subunit, which binds NADPH (nicotinamide adenine dinucleotide phosphate) and FAD (flavin adenine dinucleotide) and C-terminal regulatory domains. The catalytic domain of the protein carries out the entire enzyme reaction [6].

Severe hyperhomocysteinemia has a worldwide incidence of 1:344,000. The most common symptoms associated with this condition are ocular abnormalities such as ectopia lentis (dislocation of the eye lens), myopia and glaucoma, and skeletal abnormalities with marfanoid features such as long limbs, high-arched palate, knock knees, and sunken or funnel chest. Infants who develop homocystinuria may fail in growth and fail to gain weight at the expected rate leading to developmental delays. Furthermore, severe complications include myocardial infarction, neurodegenerative diseases, pregnancy complications [7], mental retardation, osteoporosis, and vascular complications (thromboembolism) are shown in patients that cause morbidity and mortality in untreated patients with higher homocysteine levels [2].

According to the Human Gene Mutation Database, more than 200 pathogenic mutations in the *CBS* gene and more than 40 in the *MTHFR* gene have been identified worldwide related to homocystinuria. Yet, there are only a few reports from Asian populations [5]. However, several studies have been conducted in Sri Lanka to study the allelic variants of these genes about diseases such as cardiovascular diseases; the knowledge regarding homocystinuria is very low. Thus, it is important to find out the variations of the genes associated with the disease in the Sri Lankan population, which can be used to develop novel therapeutics and enhance patient care.

## Methods

The current study was designed to detect the selected variants in *CBS* and *MTHFR* genes associated with homocystinuria in a cohort of children in Sri Lanka. Two variants from each gene, c.833 T>C/exon 8 (rs5742905) and c.19del/exon 1 (rs748695461) in the *CBS* gene and c.665C>T/exon 5 (rs1801133) and c.1286A>C/exon 8 (rs1801131) in the *MTHFR* gene, were selected based on the literature as they were common in other populations. The current study tested eight clinically and biochemically confirmed children with homocystinuria.

### Patient recruitment and sample processing

Ethical approval for the study was obtained from the Ethics Review Committee of the Faculty of Medicine, University of Colombo, Sri Lanka (EC-19-092), prior to sampling. Furthermore, additional ethical approval (LRH/DA/05/2019) was granted by the Ethics Committee of the Lady Ridgeway Hospital for Children (LRH), Colombo, Sri Lanka, as patient recruitment was conducted at the hospital.

Sample collection was carried out on eight clinically and biochemically confirmed children diagnosed between 5 and 10 years of age with homocystinuria. They are currently being followed up at their respective clinics at LRH. Genomic DNA was extracted using the QIAamp DNA Blood Mini Kit (cat. no. 51104, QIAGEN, Hilden, Germany), followed by polymerase chain reaction (PCR) for each selected variant. Sociodemographic and clinical data were obtained from the patients by filling a data collection sheet, medical records based on patient guardian interviews and patient records, and pathology reports. As per the hospital records, homocysteine and methionine levels of the patient samples were detected using high-performance liquid chromatography, and the vitamin B12 levels of patient samples were detected using gas chromatography-mass spectrometry.

### Detection of selected single-nucleotide polymorphism (SNP)

Four primer sets were designed to amplify each variant in *CBS* and *MTHFR* genes using the online NCBI/Primer-BLAST software (https://www.ncbi.nlm.nih.gov/tools/primerblast/index). PCR products were purified using the Wizard® SV Gel and PCR Clean-Up kit (Promega Corporation). Purified PCR products of two SNPs (c.833T>C in *CBS* and c.1286A>C in *MTHFR*) were subjected to SNP mini-sequencing using SNaPshot™ Multiplex Kit (Thermo Fisher Scientific, Waltham, MA, USA), supported by the in-house installed Applied Biosystems™ 3500Dx Genetic Analyzer (ThermoFisher Scientific, USA). In addition, the other two SNPs (c.19del in the *CBS* and c.665C>T in *MTHFR*) were directly sequenced using the BigDye® Terminator v3.1 kit (Thermo Fisher Scientific, Waltham, MA USA) and an Applied Biosystems™ 3500Dx Genetic Analyzer (Thermo Fisher Scientific).

## Results

Results of the SNP mini-sequencing were analysed by using Gene Marker software V2.6.4, while sequencing results were analysed using Mutation Surveyor® v4.0.9 by aligning with the Human *CBS* (GenBank accession number-NG_008938) and *MTHFR* (GenBank accession number—NG_013351.1) reference sequences at the National Centre for Biotechnology Information (NCBI). ClinVar was used to classify the variants based on the clinical significance (https://www.ncbi.nlm.nih.gov/clinvar/). Protein structure prediction for the identified variants was built using SWISS-MODEL (https://swissmodel.expasy.org/) and used P42898 (MTHFR_HUMAN) and P35520 (CBS_HUMAN) for comparison. TM align software (https://zhanglab.ccmb.med.umich.edu/TM-align/) was used for protein structure comparison and for constructing superimposed images. Table 1 summarises the status of four variants in *CBS* and *MTHFR* genes with the patients’ general information, clinicopathological characteristics, and biochemical parameters of eight patients studied in the current research work.

**Table 1:** Comparison of identified genetic variants in *CBS* with identified clinicopathological characteristics

Sample	General information				Variant status/zygosity				Clinicopathological characteristics						
	Age at diagnosis (years)	Current age at study (years)	Gender (F/M)	Family history	***CBS*** gene		***MTHFR*** gene		Ectopia Lentis	Myopia	Glaucoma	Visual impairment	Learning difficulties	Marfanoid features	Personality changes
					c.833T > C	c.19del	c.1286A > C	c.665C > T							
1	9	14	F	Not reported	Homo Wild	Homo Wild	Hetero	Homo Wild	√	√		√	√		
2	6	13	M	Consanguineous parents	Homo Wild	Homo Wild	Hetero	Homo Wild		√		√		√	
3	4	11	M	Non-consanguineous parents Grandfather had a similar eye problem Siblings	Homo Wild	Homo Mutated	Hetero	Homo Wild	√	√		√	√	√	√
4	6	20	F		Homo Wild	Homo Mutated	Hetero	Homo Wild	√	√	√	√	√	√	√
5	5	16	F	Consanguineous parents Siblings	Homo Wild	Homo Mutated	Homo Wild	Hetero	√	√		√	√	√	√
6	6	19	M		Homo Wild	Homo Mutated	Hetero	Homo Wild	√	√		√	√	√	
7	5	14	F	Consanguineous parents Siblings	Homo Wild	Homo Wild	Hetero	Homo Wild	√	√		√	√	√	
8	5	9	M		Homo Wild	Homo Wild	Hetero	Homo Wild	√	√		√	√	√	

### Results of SNP mini-sequencing

**Fig. 1:**
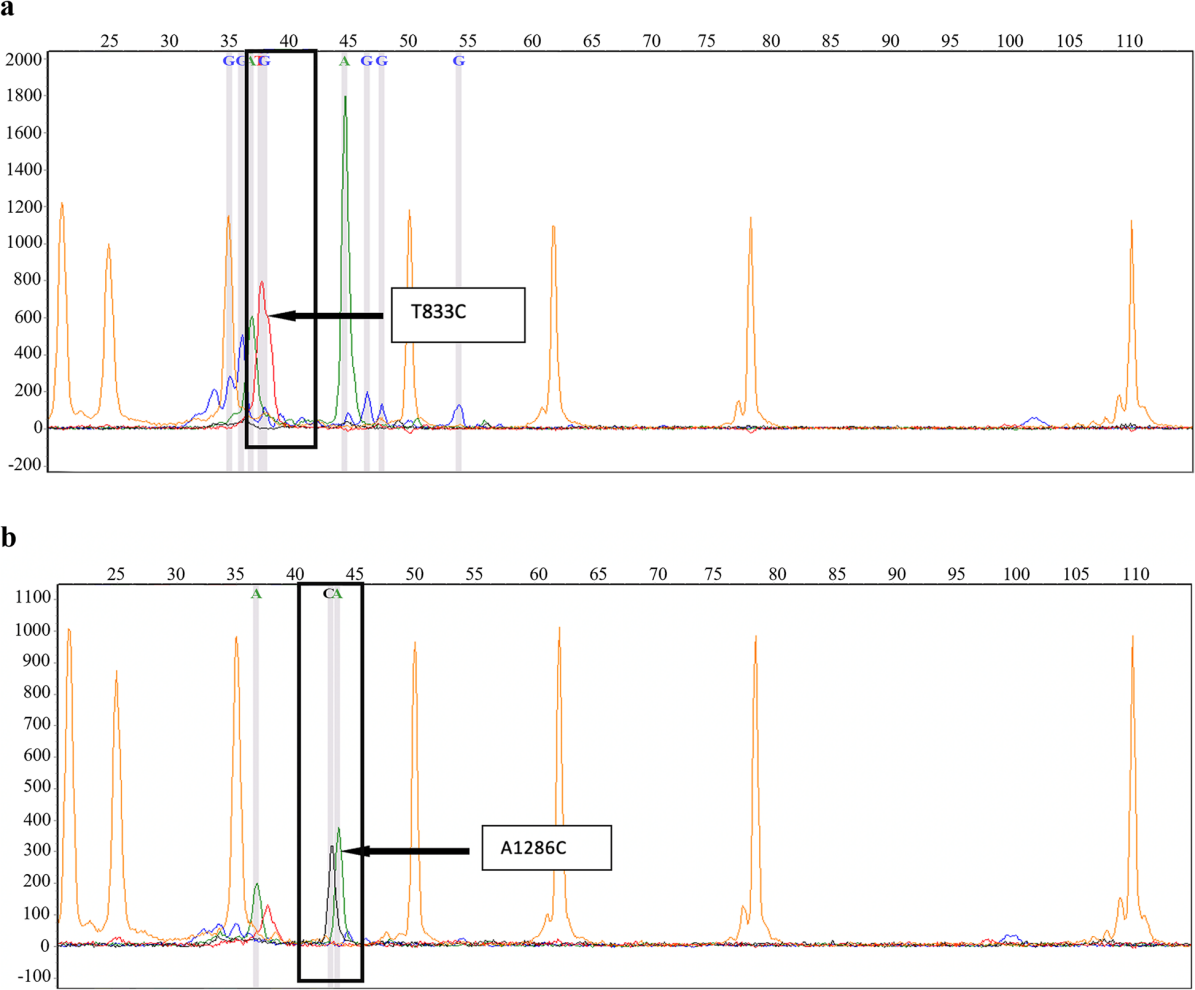
GeneMarker software image of the SNP mini-sequencing result for all samples. **a** Electropherogram is indicated the position of the expected variant c.833T>C. **b** Electropherogram is indicated as the position of the expected variant c.1286A>C

Results of the SNaPshot assay of individual samples are given in Fig. 1. Peaks of each SNP were labelled by position, followed by the type of nucleotide present at the position. The peak colours blue, green, black, red, and orange indicate the reaction of ddGTP (dideoxyguanosine 5′-triphosphate), ddATP (dideoxyadenosine 5′-triphosphate), ddCTP (dideoxycytidine 5′-triphosphate), ddTTP (dideoxythymidine 5′-triphosphate), and GeneScan 120 LIZ standards, respectively. All the electropherograms were obtained using the GeneMarker software V2.6.4.

#### c.833T>C in CBS

According to the results obtained via the GeneMarker software, all the subjected samples have one peak at the expected position of the SNP, which indicated the presence of only one nucleotide, wild-type threonine. The electropherograms of all the samples indicated the presence of homozygous wild-type condition for c.833T>C (Fig. 1a). This missense variant occurred due to the substitution of thymine by cytosine at 833rd position in exon 8 of the *CBS* gene, altering isoleucine to threonine at the 278th position in the protein (p.Ile278Thr). According to ClinVar, this variant is classified as a pathogenic variant. However, the result was further checked and confirmed for the zygosity by carrying out the Sanger sequencing for one of the representative samples.

#### c.1286A>C in MTHFR

According to the results, seven samples showed two peaks at the expected position of the SNP, indicating the presence of adenine and cytosine nucleotides (Fig. 1b). Only one peak was observed in one sample, indicating the nucleotide adenine. As a result, it was concluded that seven samples had the heterozygous condition, while one had the homozygous wild-type condition for the SNP c.1286A>C. In this missense variant, adenine is substituted by cytosine at the 1286th position in exon 8, altering glutamate to alanine at the 429th position (p.Glu429Ala) in the MTHFR enzyme. This genetic change is classified as “likely pathogenic.” Direct sequencing further confirmed these results using one representative heterozygous sample.

### Results of sequencing

#### c.665C>T in MTHFR

**Fig. 2:**
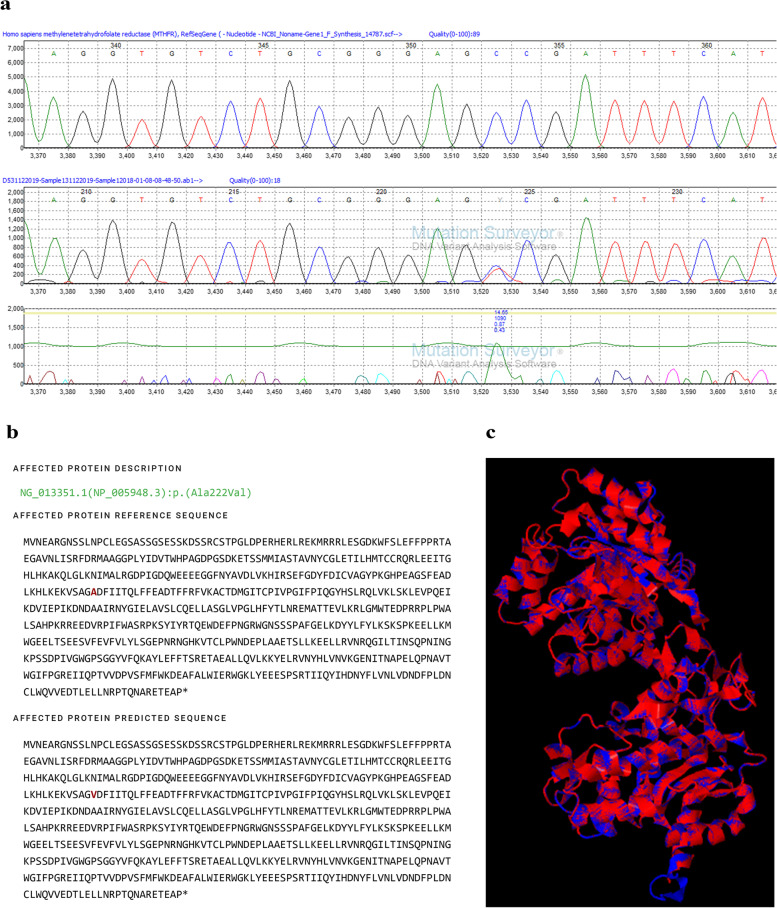
Reported missense variant, c.665C>T in MTHFR (p. Ala222Val), in exon 5. **a** Mutation Surveyor® V4.0.10 images indicating the heterozygous substitution point; respect to the reference *MTHFR* sequence and the study sample *MTHFR* sequence. **b** Protein prediction using Mutalyzer 3. **c** Superimposed image of predicted mutated protein with wild-type CBS protein (P42898 MTHFR_HUMAN), red indicates the mutated protein; blue indicates the wild-type protein

Direct sequencing was carried out for all the samples to check the SNP c.665C>T. Then, the resultant sequence was aligned with the reference sequence of the *MTHFR* gene using Mutation Surveyor® V4.0.9. According to the results, except for one sample, all the other samples did not show overlapped peaks at the expected position, which confirmed the homozygous wild-type condition for the SNP c.665C>T. In this missense variant, a cytosine at the 655th position of exon 5 of *MTHFR* is substituted by thymine; altering alanine to valine in the enzyme at the 222nd position (p.Ala222Val) is classified as a pathogenic variant with clinical interpretation as drug responsive. The resultant sample with the heterozygous condition is illustrated in Fig. 2.

#### c.19del in the CBS

**Fig. 3:**
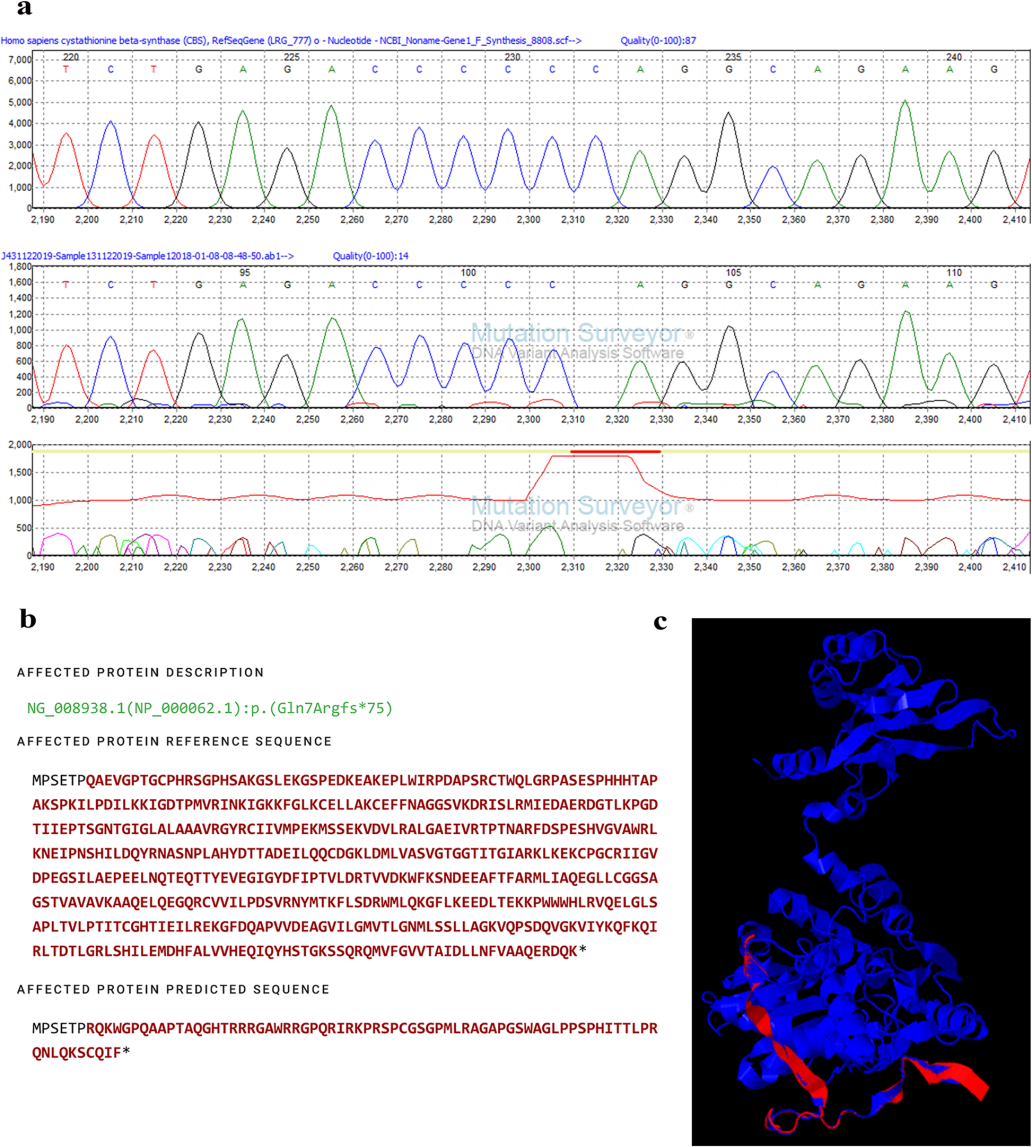
Reported nonsense variant, c.19del in *CBS* gene (p. Gln7Argfs*75*) in exon 1. **a** Mutation Surveyor® V4.0.10 images indicating the homozygous substitution point; R indicates the reference *CBS* sequence, and S indicates the study sample *CBS* sequence. **b** Protein prediction using Mutalyzer 3. **c** Superimposed image of predicted mutated protein with wild-type CBS protein (P35520 CBS_HUMAN), red indicates the mutated protein

Direct sequencing was carried out in all samples. Then, the resultant sequences were aligned with the reference sequence of the *CBS* gene using Mutation Surveyor® V4.0.9. According to the results obtained, four samples showed homozygous mutant condition for c.19del, while the other four showed homozygous wild condition for the variant. c.19del, deleterious frameshift deletion, occurs due to cytosine deletion at the 19th position of the exon 1, the coding region of the *CBS* gene, resulting in a truncated protein with lost activity (p.Gln7Argfs*75). The resultant sample with homozygous mutant condition illustrates in Fig. 3.

## Discussion

This study was mainly focused on detecting the selected variants of *CBS* and *MTHFR* genes and their association with homocystinuria. A cohort of children who are clinically confirmed to have homocystinuria were the study participants being followed up by a consultant chemical pathologist at the Lady Ridgeway Hospital for Children, Colombo.

In the current study, eight individuals were screened for the c.833T>C variant in *CBS*, and among these individuals, none of them showed the variant, and all those individuals were homozygous wild type. Hence, the assumption can be made that there is a likelihood that this mutation is not common in Sri Lankan population. When considering the world context, c.833T>C (I278T) was found in patients with homocystinuria of different ethnic backgrounds with a prevalence of 1:344,000 [8] and was reported to be associated with pyridoxine responsiveness [9]. The presence of the *CBS* c.833T>C (p.I278T) mutation was tested in 500 newborns in the Danish population and appeared to be having heterozygous state in 1.4%, corresponding to the prevalence of predicted homozygosity of 1:20,400 [10]. In addition, after screening 1133 newborns in Norway, a heterozygous state of c.833T>C was detected with a frequency rate of 0.62%, while the predicted frequency of homozygosity was 1:104,000 [11]. According to the study conducted among Czech and Slovak Republics in 2001, it was found that 5 out of 1284 screened newborns are heterozygous for c.833T>C, with a frequency rate of 0.389%, while predicting the frequency of homozygosity was 1:264,000 [12]. It was detected heterozygote frequencies of 1.5% for c.833T>C after screening 200 unrelated healthy adults in Germany and predicted homozygote frequency of 1:17,800 [13], while in the Netherlands, it was 0.4% of heterozygosity frequency and 1:250,000 of predicted homozygote frequency [14]. A total of 304,086 newborns were screened in Kuwait between January 2015 and December 2020, and only six newborn children were identified with homocystinuria with a prevalence of 1:50,000, and no c.833T>C mutation was detected [15]. The highest prevalence was reported in Qatar at 1:1800 as per a study done on 126 Qatari patients between 2016 and 2017 by Al-Dewik et al. [16]. According to Shawky et al., the prevalence of homocystinuria in the Northeast region of Cairo, Egypt, is 1.96% [17].

Studies among the Asian population related to homocystinuria are less, and few studies were conducted among Korean, Chinese, Pakistani, and Indian nationalities. Mutation analysis was done on six individuals in the Korean population [18], and research conducted among individuals of a Chinese family [19] did not identify the c.833T>C mutation. In 2011, c.833T>C and c.1006C>T in the *CBS* gene were detected in Hong Kong patients with homocystinuria*.* However, those are the only reported mutations in the *CBS* gene in the Chinese population [20] and were also the first time that c.833T>C was reported in the Asian population. Kaur R. et al. conducted a study on 16 northern Indian children in 2020 who did not identify with the c.833T>C mutation [21]. In 2021, Wasim M. et al. conducted a mutation analysis on 429 patients with an intellectual disability from northern areas of Punjab, Pakistan. Only nine patients were identified with homocystinuria, and none had c.833T>C mutation [22]. A study on three patients in a Pakistani consanguineous family with symptoms in 2021 confirmed homocystinuria was not detected with c.833T>C mutation [23]. Although the prevalence of c.833T>C is likely less among the Asian population, but it is the most prevalent mutation worldwide in the *CBS* gene related to homocystinuria, thus, it is important to find out the presence and prevalence of this mutation in Sri Lanka.

Among the 485 SNPs recorded in the NCBI SNP database, at least 40 variants in the *MTHFR* gene have been identified in people with homocystinuria. Out of them, c.665C>T and c.1286A>C are the two major variants in the *MTHFR* gene responsible for increasing homocysteine levels in the blood [24]. According to previous studies, the presence of c.665C>T variant in either a homozygous or a compound heterozygous form with c.1286A>C in the *MTHFR* gene leads to the severe reduction of the MTHFR enzyme levels, causing an elevated level of homocysteine in the blood [25]. In the current study, none of the patients shows the co-existence of c.665C>T and c.1286A>C in the *MTHFR* gene. For c.665C>T, the enzyme activity of heterozygous and homozygous mutant individuals is 67% and 25%, respectively, compared to the wild-type ones. However, c.1286A>C, the enzyme activity of heterozygous and homozygous mutant individuals, is 83% and 61%, respectively, which of the wild-type subjects [25]. A study done on the geographic and ethnic distribution of the c.665C>T variant in newborns showed that homozygous mutant type (TT genotype) was prevalent in Northern China (20%), Southern Italy (26%), and Mexico (32%) where the frequency of the T allele in newborns of Asian origin was low [26]. Alternatively, a study done in Sri Lanka by Amarakoon et al., to check the association of the variants with hyperhomocysteinemia (high homocysteine levels), showed that 89.9% of individuals were homozygous wild type, while there were individuals with heterozygous and homozygous mutants for c.665C>T with the prevalence rate of 3.8% and 6.3%, respectively. In addition, 41.8% of individuals were homozygous wild type, and the rest were heterozygous and homozygous mutants for the c.1286A>C in the *MTHFR* gene with rates of 32.7% and 25.5%, respectively [27]. According to another study done in Sri Lanka by Perera et al., *MTHFR* variants for ischemic heart disease (IHD) showed prevalence rates of 72.8%, 24.7%, and 2.5% for CC (homozygous wild type), CT (heterozygous mutant), and TT (homozygous mutant) genotypes in c.665C>T, respectively, showing CC genotype was the predominant genotype among IHD patients and controls. In contrast, the TT genotype was detected only among very few IHD patients. Prevalence rates of c.1286A>C were 50% for the AA (homozygous wild type), 37.3% for the AC (heterozygous), and 12.7% for CC (homozygous mutant) genotypes for the whole study population, where the AA genotype was the predominant genotype among controls, and AC genotype was the predominant genotype among IHD patients [28]. According to the analysis of the current study on *MTHFR* gene variants, seven samples showed heterozygous mutant status for c.1286A>C, while one patient had the heterozygous mutant for c.665C>T condition. Hence, the frequency of c.1286A>C was 87.5%, and c.665C>T was 12.5%, indicating that the c.1286A>C SNP was much more likely to be prominent than the SNP c.665C>T among patients with homocystinuria in the studied cohort. Thus, the presence of c.665C>T variant in the *MTHFR* gene dissociates the active dimer into monomers, losing the MTHFR enzyme’s FAD-binding capacity, which leads to hypomethylation and increases the homocysteine levels [29].

According to the direct sequencing results, the presence of c.19del variant in the *CBS* gene was shown in four samples with the homozygous state, while the other four samples did not show this variant. Thus, the frequency for this variant is 50% in the studied cohort, showing a significant appearance of the variant in patients with homocystinuria in Sri Lanka. According to the ExAC browser, c.19del was previously reported in a heterozygous state in two South Asian alleles, which was not found to be related to homocystinuria. In a study done by Kaur R. et al., in 2020, out of sixteen children, four patients were confirmed to have c.19del in North Indian children with homocystinuria [21]. However, these frequencies will not reflect the actual frequency distributions of these variants in Sri Lanka, as the number of samples studied in this preliminary research is insufficient to conclude a result. Thus, a randomly selected larger cohort needs to be studied in the future to determine the prevalence of the above variants in children with homocystinuria in Sri Lanka.

According to the overall result shown in Table 1, indicating the fact that all the children have symptoms more or less similar associated with homocystinuria, where four children (S003, S004, S005, and S006) had comparatively severe symptoms than the other patients. Patients S003 and S004 are children of a non-consanguineous marriage, who were presented with severe clinical symptoms such as ectopia lentis, myopia, glaucoma, visual impairment, learning difficulties, marfonoid features, and personality changes, having elevated plasma total homocysteine and methionine levels, and were detected for having c.19del variant in homozygous condition. Since this is a recessive inherited disorder, where both patients in this family are homozygous for the condition, their parents should be heterozygous, asymptomatic, and carriers. According to the data obtained from the patients, they have a suspected family history of this disease where their paternal grandfather had similar eye problems. This paternal pedigree likely shares this variant over a few generations as heterozygous carriers. Since parents are not related, analysing the pedigrees of families (paternal and maternal), if possible, is required to conclude the inheritance and origin of this mutation.

Another two siblings, S005 and S006, were diagnosed with homocystinuria based on the detection of elevated blood homocysteine and severe clinical symptoms such as ectopia lentis, myopia, visual impairment, learning difficulties, and marfonoid features. They are children of consanguineous parents and had no evidence of family history for this condition. S005 and S006 are siblings who are homozygous for c.19del in the *CBS* gene. Due to the recessive inheritance of the disease and parents being related, the genetic variant is expected to be shared and formed over a few generations. As these two children have two different SNP conditions for the gene, *MTHFR* pedigree analysis is necessary to confirm the inheritance. However, patient S005 has the heterozygous condition for SNP c.665C>T, and S006 has the heterozygous condition for SNP c.1286A>C. According to the gender difference, different effects can occur in homocysteine levels in normal and heterozygous states. Females are found to have lower homocysteine levels than males. This difference is mainly due to the protective effect of oestrogen and other physiological factors in women. The severity of symptoms and plasma homocysteine levels are more prominent in the homozygous TT genotype in c.665C>T [30]. However, no such difference was seen between the heterozygous A>C patients and heterozygous C>T when comparing the symptoms in the studied cohort. Hence, the effect of the variant might be almost the same according to the results obtained. However, the sample size is comparatively small to conclude the difference between symptoms and genetic conditions.

All samples with c.19del in the *CBS* gene were from the southern province in Sri Lanka. Except for one, all samples from the southern province shared heterozygosity for the c.1286A>C variant in the *MTHFR* gene. Only one has the heterozygous condition for c.665C>T, while others have the homozygous wild type in the southern province. They might be likely to share a common genetic basis. So, studying these families’ pedigrees would significantly impact the origin and inheritance of these mutations.

The primary purpose of treatment for homocystinuria is to reduce plasma homocysteine levels, normalise methionine levels, and alleviate a patient’s clinical symptoms. Administration of betaine of 2–9 g/d in children to facilitate an alternate pathway for methylation reactions, then hydroxycobalamin of 1–2 g/d (vit. B12) as a cofactor for methionine synthase, folate of 400 mg/d to increase residual MTHFR function and pyridoxine (vit. B6) to maximise the transsulfuration pathway as a cofactor for CBS, and riboflavin (vit. B2) for the flavin component of the MTHFR enzyme are some treatment options for homocystinuria. There is only limited success with methionine supplements and MTHFR enzyme replacements [31]. According to murine studies, it was identified that the prophylactic treatment with high-dose folate and betaine in pregnant and lactating women benefits the high-risk families [32]. As per Table 2, the onset homocysteine levels of the patients have been considerably higher than the optimum range (4.6–8.1 μmol/L). However, after the treatment of betaine and pyridoxine, the levels have dropped comparatively but have not fallen into the normal range. After treatments, the homocysteine levels of patients S001 and S006 have significantly dropped compared to the other patients and have become closer to the optimum range.

**Table 2:** Comparison of homocysteine, methionine and vitamin B12 levels of the patients before and after the treatments

Sample no.	Biochemical parameters as per hospital records					Treatment
	Homocysteine level (μmol/L) (4.6–8.1 μmol/L)		Methionine level (μmol/L) (6–60 μmol/L)		Vitamin B12 level (pmol/L) (140–650 pmol/L)	
	Before treatment	After treatment	Before treatment	After treatment		
S001	261	26	66	17	184	Pyridoxine Betaine
S002	292	296	227	700	162	Pyridoxine
S003	318	168	177	N/A	216	Betaine
S004	373	213	79	N/A	160	Betaine
S005	345	132	59	1261	145	Betaine
S006	235	21	745	1817	203	Betaine
S007	366	201	91	128	198	Pyridoxine
S008	203	218	220	471	222	Pyridoxine

On the contrary, most of the patients have noticeably higher methionine levels than the normal range (6–60 μmol/L) initially, and in some patients, their methionine levels have further increased after treatment of betaine which was given to reduce the homocysteine levels and to aid the methylation reaction. Vitamin B12 levels of a normal person vary from 140–650 pmol/L. Furthermore, all the patients had vitamin B12 within the above range when this study was conducted. Vitamin B12 acts as a cofactor for the enzyme MTHFR, and if this vitamin is deficient, it affects the activity of the enzyme interfering with the metabolic pathway and causing a high amount of homocysteine in blood or urine. Vitamin B12 levels of these patients were within the normal range at the time of the study, indicating that the disease could not be due to the vitamin B12 deficiency but to the mutations of genes that aid the methylation reaction.

## Conclusion

This is the first study to deliver a genetic characterisation of homocystinuria in Sri Lanka to the best of our knowledge. When comparing the results of this study with the other studies done worldwide, the results merely gave a pattern among the Sri Lankan population. As mentioned, the variant conditions did not follow a constant distribution but can be varied depending on different sociodemographic factors. Hence, further studies are needed with a larger cohort to confirm these conditions associated with the disease. Furthermore, it is important to study the correlation of both of these variants for the occurrence of the disease and its symptoms. Further studies are needed with a larger cohort of patients to assess the effect of both of these variants.

## Data Availability

The datasets generated, used and/or analysed during the current study, are available from the corresponding author on reasonable request.

## Abbreviations

CBS: Cystathionine β-synthase
FAD: Flavin adenine dinucleotide
MTHFR: Methylenetetrahydrofolate reductase
MTR-5: Methyltetrahydrofolate-homocysteine methyltransferase
MTRR-5: Methyltetrahydrofolate-homocysteine methyltransferase reductase
NADPH: Nicotinamide adenine dinucleotide phosphate
